# Evaluation of S100A8/A9 and neutrophils as prognostic markers in metastatic melanoma patients under immune-checkpoint inhibition

**DOI:** 10.1016/j.tranon.2024.102224

**Published:** 2024-12-18

**Authors:** Yasmin F Melzer, Nadine L Fergen, Christian Mess, Julia-Christina Stadler, Glenn Geidel, Ysabel A Schwietzer, Julian Kött, Klaus Pantel, Stefan W Schneider, Jochen Utikal, Ewa Wladykowski, Sabine Vidal-y-Sy, Alexander T Bauer, Christoffer Gebhardt

**Affiliations:** a Department of Dermatology and Venereology, University Medical Center Hamburg-Eppendorf (UKE), Hamburg, Germany; b Fleur Hiege Center for Skin Cancer Research, University Medical Center Hamburg-Eppendorf (UKE), Hamburg, Germany; c Department of Tumor Biology, University Medical Center Hamburg-Eppendorf (UKE), Hamburg, Germany; d Skin Cancer Unit, German Cancer Research Center (DKFZ), Heidelberg, Germany; e Department of Dermatology, Venereology and Allergology, University Medical Center Mannheim, Ruprecht-Karl University of Heidelberg, Mannheim, Germany; f DKFZ Hector Cancer Institute at the University Medical Center Mannheim, Mannheim, Germany

**Keywords:** Melanoma, Immune-checkpoint inhibitors, Neutrophils, S100A8/A9, Inflammation, Tumor progression, Biomarker

## Abstract

Immune-checkpoint inhibitors (ICIs) have revolutionized melanoma treatment, yet approximately half of patients do not respond to these therapies. Identifying prognostic biomarkers is crucial for treatment decisions. Our retrospective study assessed liquid biopsies and tumor tissue analyses for two potential biomarkers: danger-associated molecular pattern (DAMP) S100A8/A9 and its source, neutrophils. In 43 metastatic unresected stage III/IV melanoma patients, elevated serum levels of S100A8/A9 and neutrophils before and during ICI treatment correlated with worse outcomes. Furthermore, in 113 melanoma patients, neutrophil expression in the tumor microenvironment (TME) was associated with relapse and reduced survival. Measuring S100A8/A9 and neutrophils could enhance immunotherapy monitoring by predicting impaired clinical outcomes and non-response to ICIs. Serum S100A8/A9 levels and neutrophil counts at baseline (T0) and during treatment (T3) correlated with reduced progression-free survival (PFS). Elevated S100A8/A9 levels at T0 and T3 negatively impacted overall survival (OS). Notably, neutrophil infiltration was more prevalent in primary melanomas than in nevi and metastases, and its presence in primary melanomas was linked to poorer survival. S100A8/A9 serum levels, neutrophil counts, and tumor-associated neutrophil infiltration represent promising biomarkers for predicting treatment response and clinical outcomes in melanoma patients receiving ICIs.

**Significance:**

These findings underscore the critical need for reliable biomarkers in melanoma research, particularly for predicting responses to immune-checkpoint inhibitors (ICIs). Identifying S100A8/A9 levels and neutrophil infiltration as potential indicators of treatment outcomes offers valuable insights for personalized therapy decisions. By enhancing monitoring and prognosis assessment, these biomarkers contribute to refining treatment strategies, ultimately improving patient care and outcomes. This research bridges gaps in understanding melanoma response mechanisms and highlights avenues for further investigation into immune-related markers, fostering advancements in precision medicine for melanoma patients.

## Highlights

• Increased levels of circulating S100A8/A9 during ICI and accumulation of TANs impair clinical outcome of metastatic melanoma.
• These findings underline the relevance of neutrophils and S100A8/A9 as drivers of melanoma progression.
• In clinical practice, serum S100A8/A9 and neutrophil counts could serve as follow-up markers during ICI.
• Our study warrants for testing therapies targeting S100A8/A9 and other NET-associated factors in metastatic melanoma.

## Graphical abstract

Graphical abstract. A) The aim of our study was to provide evidence for S100A8/A9 and neutrophils as valuable prognostic and predictive biomarkers for tumor growth in melanoma. Our research intention was to explore the link between circulating and tumor-resident factors. Identifying additional biomarkers could constitute the foundation for effective ICIs monitoring and novel combination therapies. B) Our study consisted of two parts: 1) We analyzed liquid biopsies and measured serum S100A8/A9 concentrations at two different time points via ELISA (MRP8/14) in a cohort of 43 stage III and IV melanoma patients treated with ICIs. We obtained neutrophil count in peripheral blood from routine clinical laboratory analysis. Using Kaplan Meier curves, we estimated the impact of increasing S100A8/A9 and neutrophil levels during therapy on PFS and OS. 2) We performed immunofluorescence staining of neutrophils with anti-CD15 and Alexa 594 on 292 tissue samples including melanocytic nevi, primary melanomas and metastases from 113 patients of different stages. The tissue punches were collected on tissue micro-arrays. We compared neutrophil accumulation in the three different tumor types and analyzed the impact of neutrophil infiltration on survival.

**Figure:**
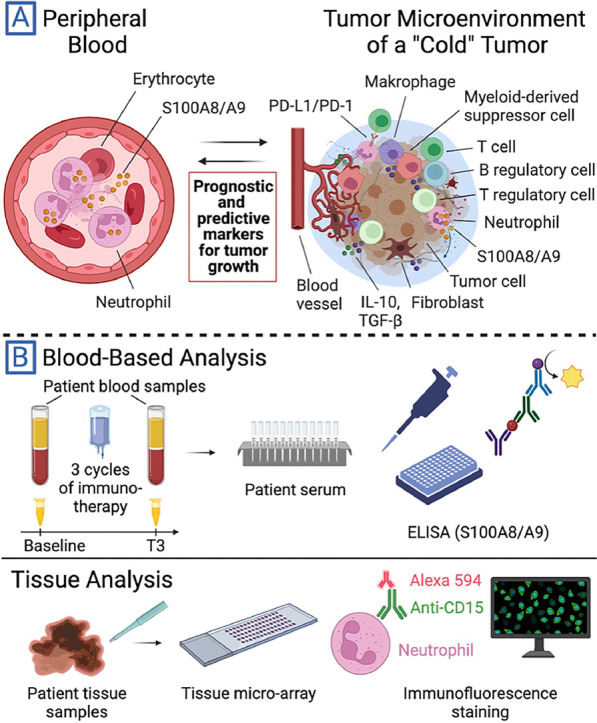

## Introduction

Since the advent of immunotherapy, unprecedented successes have been achieved in melanoma therapy by application of anti-programmed cell death protein 1 (PD-1) antibodies (pembrolizumab and nivolumab) and anti-cytotoxic T-lymphocyte protein 4 (CTLA-4) antibodies (ipilimumab). However, treatment failure or severe immune-related adverse drug reactions do occur in about 50 % of patients [48,49]. These limited response rates give rise to the quest for biomarkers in order to predict clinical outcomes during therapy.

Response to treatment was found to be strongly impacted by the immune contexture of tumors. Being based on the blockade of immune escape through T cell reactivation, ICIs especially fail in T cell-depleted tissues, which have been titled “cold” tumors [11]. T cell exclusion is caused by myeloid-derived suppressor cells (MDSCs), cancer-associated fibroblasts (CAFs) and, remarkably, neutrophils [1,4]. The latter were shown to modify inflammatory pathways and inhibit T cell activity through PD-1 expression [25,28]. Neutrophils even promote T cell apoptosis, e.g. by ROS production [31]. Interestingly, activated neutrophils secrete very large amounts of the damage-associated molecular pattern molecule (DAMP) S100A8/A9, which interferes with immune-regulatory processes and exerts proinflammatory effects [[12], [13], [14],^18^]. Neutrophils and S100A8/A9 seem to define the prognosis of melanoma patients due to their impairment of T cell functions. Consequently, both neutrophils and S100A8/A9 qualify as potential biomarkers to predict patient outcome and response to ICIs [15,46].

S100A8/A9 was proposed as a marker of inflammatory dysregulation in cancer patients in previous research projects [2]. A positive association with malignant melanoma progression was confirmed [15,20,46,47]. Besides being secreted by myeloid and other vascular cells, monocytes and tumor cells, S100A8/A9 is released by neutrophils upon a programmed cell-death process called NETosis [19,24]. The role of neutrophils in cancer has been extensively studied [17,32]. In multiple solid tumor entities, high levels of circulating neutrophils as well as an abundance of tumor-associated neutrophils (TANs) were associated with rapid tumor growth [34,36,37,51].

The predictive value of circulating serum S100A8/A9 and neutrophils as well as TANs as follow-up parameters during immunotherapy needs to be further investigated. Here, we conducted ELISA experiments in order to quantify S100A8/A9 and neutrophil count in the peripheral blood of 43 patients before and during treatment with ICIs. For additional analyses of TANs, we performed immunofluorescence stainings of tumor tissues from 113 melanoma patients.

## Patients and methods

### Blood-based analysis

#### Patient cohort and clinical data

Peripheral blood samples were obtained from 43 metastatic melanoma patients receiving ICIs at the Skin Cancer Center (University Medical Center Hamburg, Germany). This study was approved by the local ethics committee (approval code: PV5392) and we performed the collection of blood, tissue and clinical data upon the patients’ written informed consent. Patients were included if they had unresectable stage III or stage IV melanoma according to the AJCC 2017 classification and were treated with either pembrolizumab, 10 mg per kg body weight every 3 weeks or both nivolumab, 1 mg per kg body weight, and ipilimumab, 3 mg per kg body weight every 3 weeks, or nivolumab only. Other inclusion criteria were: above 18 years of age and no specific melanoma therapy during the previous 28 days. All histologic types of melanoma, including mucosal and uveal melanoma, were eligible for analyses. Patients with autoimmune disease, hepatitis B or C, HIV, pregnancy, or concomitant systemic therapy for melanoma were excluded from the study. Treatment efficacy was assessed by contrast-enhanced CT/MRT/PET-CT every 12 weeks after the first administration of therapy. Clinical responses were defined based on immune-related response criteria and indicated as complete response (CR), partial response (PR), stable disease (SD) and progressive disease (PD). Depending on their best overall response, patients were defined as responders (showing CR, PR, and SD) and non-responders (PD).

#### Analysis of peripheral blood samples

Peripheral blood was taken before the first administration of ICIs (baseline) and before the fourth perfusion (cycle 4 = T3). Serum was collected after centrifugation of blood samples for 10 min at 1,000 g, aliquoted, and stored at −80°C. S100A8/A9 levels were determined in patient serum anticoagulated with ethylenediaminetetraacetic acid (EDTA) via ELISA, which detects the MRP8/14-Antigen (EK-MRP8/14, Bühlmann), according to the manufacturer's instructions. Cell counts for leukocyte subpopulations (white blood count, WBC) were measured in patients’ venous blood by routine clinical laboratory analysis using a Sysmex XE-5000 analyzer (Sysmex).

### Tissue-based analysis

#### Patient cohort and clinical data

For tumor microenvironment analysis, we stained tissue samples from 113 unresected metastatic melanoma patients from University Medical Center Mannheim, Germany (ethic approval code: 2010-318N-MA). A total of 292 tissue punches of 113 unresected melanoma patients assembled on tissue micro-arrays (TMA) were included in this study. The cohort was monitored for 7 years and disease trajectories (e.g., blood analyses and stagings) were documented. If cutaneous metastasis occurred, a biopsy was taken from the metastatic tissue and the sample was processed and archived for staining.

#### Immunofluorescence staining

Tissue punches of melanocytic nevi, primary tumors and metastases were analyzed (see Supplements Table S1). Immunofluorescence staining was conducted on sections of formalin-fixed paraffin-embedded tumor tissues on TMA. We included 44 nevi, 86 primary tumors and 162 metastases. Neutrophils were stained using an anti-CD15 antibody (ab135377, Abcam) and a red fluorescent secondary antibody, Alexa 594 (A-11072, Invitrogen). Also, a blue nucleus staining with 4′,6-diamidino-2-phenylindole (DAPI, 10236276001, Roche) was performed. A negative control without the first antibody and a verified positive control were stained to avoid false positive or negative results.

#### Analysis of stained tissue samples

The quantification of neutrophils was conducted using immunofluorescence microscopy (Zeiss Axio Observer.Z1, 20x). The cell numbers determined by hand, using a counting tool in Image J software (version 1.53c), were divided by the measured tissue areas (in mm^2^) in order to account for differences in tissue punch sizes (see Supplements Table S1).

### Statistical analysis

All statistical calculations were performed with R (version 4.1.1) and GraphPad PRISM (version 9). One-way analyses of variance on ranks (Kruskal-Wallis test) with Wilcoxon-Mann-Whitney post-hoc tests were used to compare tumor-associated neutrophil counts. P-values less or equal than 0.05 were considered statistically significant. Progression-free survival (PFS) was defined as the time from treatment start or inclusion to the timepoint of disease progression according to CT/MRT/PET-CT imaging. Overall survival (OS) was calculated as the time from the start of ICIs treatment until death due to any cause and could not be determined for patients in the tissue expression analysis because hardly any deaths had occurred by the end of the observation period. OS and PFS were represented as Kaplan–Meier curves and cut-off values were calculated by optimizing the log-rank statistics.

## Results

### Blood-based analysis

#### Patients characteristics

The serum analysis included 43 melanoma patients receiving ICIs (nivolumab + ipilimumab (*n* = 26), nivolumab (*n* = 5) or pembrolizumab (*n* = 11)). The median age was 60.6 years and the cohort comprised 30 males (69.8 %) and 13 females (30.2 %) (see Table 1). Distant metastases were found in 41 patients (91 %) and 4 patients (9 %) had unresectable stage III disease. Among the cohort were 3 metastatic uveal melanoma and 3 mucosal melanoma. Determined by their best overall response to therapy, 11 patients achieved CR (25.6 %), 16 patients showed PR (37.2 %) and 5 patients had SD (11.6 %). For the evaluation of biomarkers, these patients were summarized as responders, whereas 11 individuals who showed PD (25.6 %) were defined as non-responders.

**Table 1:** Clinical characteristics of patients for blood-based analyses

Clinical characteristics	(*n* = 43)
**Sex**	
f	13 (30.2 %)
m	30 (69.8 %)
**Age**	
median (sd)	60.6 (18.4)
**AJCC Stage**	
IIIC	3 (7.0 %)
IV	39 (90.7 %)
IVA	1 (2.3 %)
**Staging**	
Complete Response (CR)	11 (25.6 %)
Partial Response (PR)	16 (37.2 %)
Stable Disease (SD)	5 (11.6 %)
Progressive Disease (PD)	11 (25.6 %)
**Response**	
Responder (CR, PR, SD)	32 (74.7 %)
Non-responder (PD)	11 (25.6 %)

Abbreviations: f: female. m: male. sd: standard deviation. AJCC: American Joint Committee of Cancer. n: number. CR: complete response. PR: partial response. SD: stable disease. PD: progressive disease.

#### Serum S100A8/A9 increase during ICIs treatment: impact on survival

**Fig. 1:**
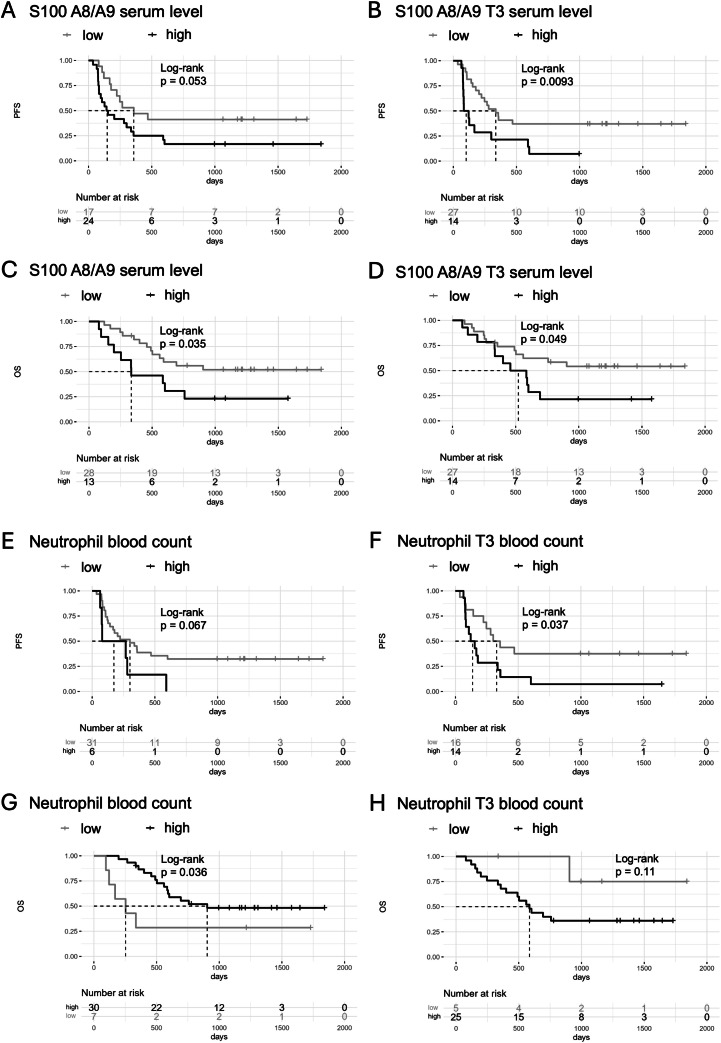
Univariate survival analyses: impact of baseline serum levels and T3 serum levels of S100A8/A9 and neutrophils on progression-free survival (PFS) and overall survival (OS). Kaplan-Meier curves are shown for the melanoma patients in the systemic study. A) For high S100A8/A9 serum levels before therapy initiation, there was a negative effect on PFS of the melanoma patients. In this analysis, the "number at risk" at day 0 for baseline serum S100A8/A9=low was 17 patients and for baseline serum S100A8/A9=high, it was 24 patients. The cut-off value was 2864.35 ng/ml. B) In the analysis at T3, high serum S100A8/A9 levels had a negative effect on PFS. Here, the "number at risk" at day 0 for T3 serum S100A8/A9=low was 27 patients and for T3 serum S100A8/A9=high, it was 14 patients. The cut-off value was 3770.73 ng/ml. C) For high baseline serum S100A8/A9 levels, there was a negative effect on OS of melanoma patients. In this analysis, the "number at risk" at day 0 for baseline serum S100A8/A9 =low was 28 patients and for S100A8/A9 =high, it was 13 patients. The cut-off value was 4499.26 ng/ml. D) Low serum S100A8/A9 levels at T3 had a positive effect on the OS of melanoma patients. In this analysis, the "number at risk" at day 0 for the T3 serum neutrophils=low was 27 patients and for T3 serum neutrophils=high, it was 14 patients. The cut-off value was 3770.73 ng/ml. E) In this analysis regarding PFS, there was no significant difference between patients with high neutrophil count in peripheral blood at baseline and those melanoma patients who had low systemic neutrophil count. The "number at risk" at day 0 for baseline neutrophil count in peripheral blood=low was 31 patients and for baseline count in peripheral blood=high, it was 6 patients. The cut-off value was 6.02/nl. F) For a high count of neutrophils at T3, there was a negative effect on PFS of melanoma patients. The "number at risk" at day 0 was 16 patients for T3 neutrophil count in peripheral blood=low and 14 patients for T3 neutrophil count in peripheral blood=high. The cut-off value was 4.35/nl. G) For high neutrophil count in peripheral blood at baseline, there was a positive effect on OS of the melanoma patients. In this analysis, the "number at risk" at day 0 for baseline neutrophil count in peripheral blood=low was 7 patients and for baseline neutrophil count in peripheral blood=high, it was 30 patients. The cut-off value was 3.84/nl. H) In the analysis with the endpoint OS, high T3 neutrophil count in peripheral blood were associated with no significant impact on survival. In this analysis, the "number at risk" at day 0 for T3 neutrophil count in peripheral blood=low was 5 patients and for T3 neutrophil count in peripheral blood=high, it was 25 patients. The cut-off value was 3.17/nl.

For biomarker testing, we aimed at exploring the value of S100A8/A9 as follow-up biomarker with maximal clinical significance during therapy. Therefore, we quantified S100A8/A9 serum concentrations at baseline (T0, before the start of therapy) and again before the fourth perfusion of ICIs (T3). We then conducted a univariate survival analysis regarding the endpoints PFS (see Fig. 1A and B) and OS (see Fig. 1C and D). A lower serum level of S100A8/A9 was significantly associated with prolonged PFS (cut-off-value 2864.35 ng/ml, *p* < 0.05). The analysis regarding OS demonstrated that increased concentrations of S100A8/A9 correlated with reduced OS. Thus, high concentrations of S100A8/A9 in the serum had an unfavorable impact on the prognosis of the patient.

#### Circulating neutrophil increase during ICIs treatment: impact on survival

Secondly, we performed Kaplan-Meier analyses of two time points, T0 (baseline) and T3 (before the fourth infusion of ICIs) with regard to the effect of neutrophil count in peripheral blood on PFS (see Fig. 1E and F) and OS (see Fig. 1G and H). The analysis regarding PFS almost reached significance and showed a tumor-promoting effect of circulating neutrophils (*p* = 0.067). In line, if the serum level of neutrophils was significantly increased at T3, PFS was reduced. By contrast, high count in peripheral blood had a favorable impact on OS at baseline and no impact on OS at T3. In summary, a low neutrophil count in peripheral blood was associated with prolonged PFS, which suggests a negative effect of neutrophils on tumor progression.

### Tissue analysis

#### Patients characteristics

The tissue expression analysis included 113 unresected cutaneous melanoma patients (see Table 2). The median age was 65.1 years and the cohort contained 73 males (64 %) and 41 females (36 %). Distant metastases were found in 8 patients (91 %) and 29 patients (26 %) had unresectable stage III disease; 60 patients were considered stage II (54 %) and 14 patients were diagnosed with stage I malignant melanoma (13 %). A BRAF-mutation V600E was confirmed in 10 patients (9 %).

**Table 2:** Clinical characteristics of patients for tissue analyses

Clinical characteristics	(*n* = 113)
**Sex**	
f	41 (36.3 %)
m	72 (63.7 %)
**Age**	
median (sd)	65.1 (13.0)
**AJCC Stage**	
I	14 (12.6 %)
II	60 (54.1 %)
III	29 (26.1 %)
IV	8 (7.2 %)
**BRAF-Mutation**	
V600E	10 (8.8 %)
WT Not specified	8 (7.1 %) 95 (84.1 %)

Abbreviations: sd: standard deviation, BRAF: B Rapidly Accelerated Fibrosarcoma, V: Valin, E (one letter code): glutamic acid, WT: Wild Type.

#### Neutrophil infiltration of different tumor types

**Fig. 2:**
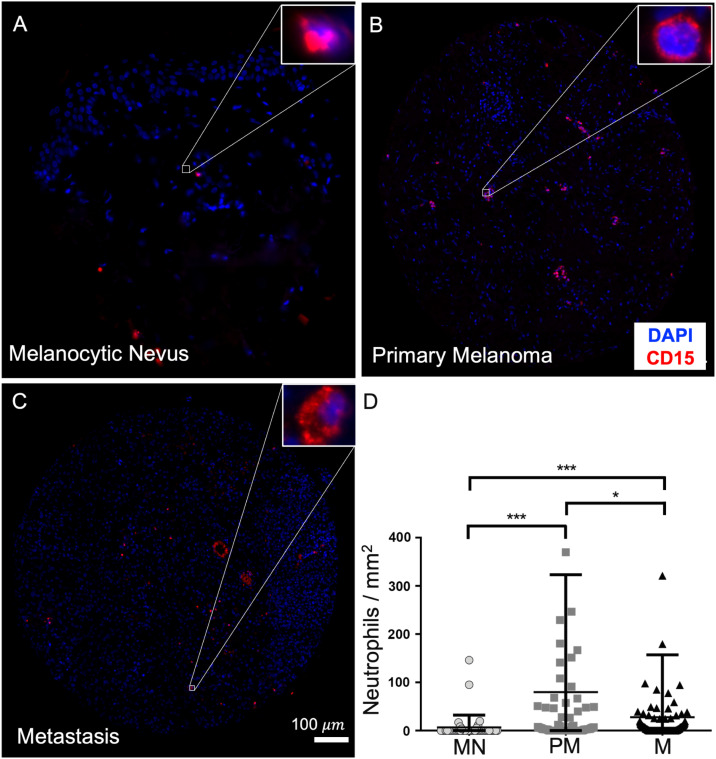
Immunofluorescence stainings and analyses of an average low neutrophil infiltrated nevus (A) and an average high infiltrated primarius (B) and metastasis (C). Statistical analysis of all cell counts obtained (D). Shown are microscopic overview images of the tissue punches of a nevus (TMA 1, punch L17), a primary melanoma (TMA 2.1, punch A11), and a metastasis (TMA 2.2, punch E8) as representation of the respective tissue types. The tissue samples were stained with an anti-CD15 antibody in conjunction with the secondary antibody Alexa 594 to visualize neutrophil granulocytes in red color. A single neutrophil from each punch is shown magnified. Blue nuclear counterstaining was performed with DAPI. Graphically, the neutrophil counts of the total 44 nevi, 86 primaries, and 162 metastases examined are listed in D. Significance levels: *: *p* < 0.05; ***: *p* < 0.001. Abbreviations: MN: melanocytic nevus, PM: primary melanoma, M: metastasis.

For comparison of neutrophil infiltration in the respective tissues, representative example images of the melanocytic nevi, primary melanomas and metastases examined, as well as the corresponding statistical data analysis, are depicted in Fig. 2. On average, 6 neutrophils were found in melanocytic nevi samples and 68 neutrophils in primary melanomas (measured as number of cells per normalized punch area in each case). Thus, more than 11x more neutrophils were found in primaries than in melanocytic nevi (see Fig. 2A, B, D). Metastases contained an average of 23 neutrophils and hence about 4x more neutrophil granulocytes than melanocytic nevi (see Fig. 2A, C, D). Primary melanomas contained on average about 3x more neutrophils than metastases (see Fig. 2B–D). By performing one-way ANOVA analyses as shown in Fig. 2D, we confirmed that the number of neutrophils was significantly lower in melanocytic nevi versus in primary melanomas (*p* < 0.001). Between melanocytic nevi and metastases, neutrophil counts were likewise significantly different. They were lower in melanocytic nevi (*p* < 0.001). The number of infiltrating neutrophils in primary melanomas versus in metastases was significantly smaller in metastases (*p* < 0.05). In summary, significantly fewer neutrophils were detected in melanocytic nevi compared to primary melanomas and metastases. Consequently, in the cohort of patients considered, the fewest neutrophils were identified in melanocytic nevi and the most neutrophil granulocytes were present in primary melanomas, on average.

#### High infiltration of tumor-associated neutrophils: impact on survival

**Fig. 3:**
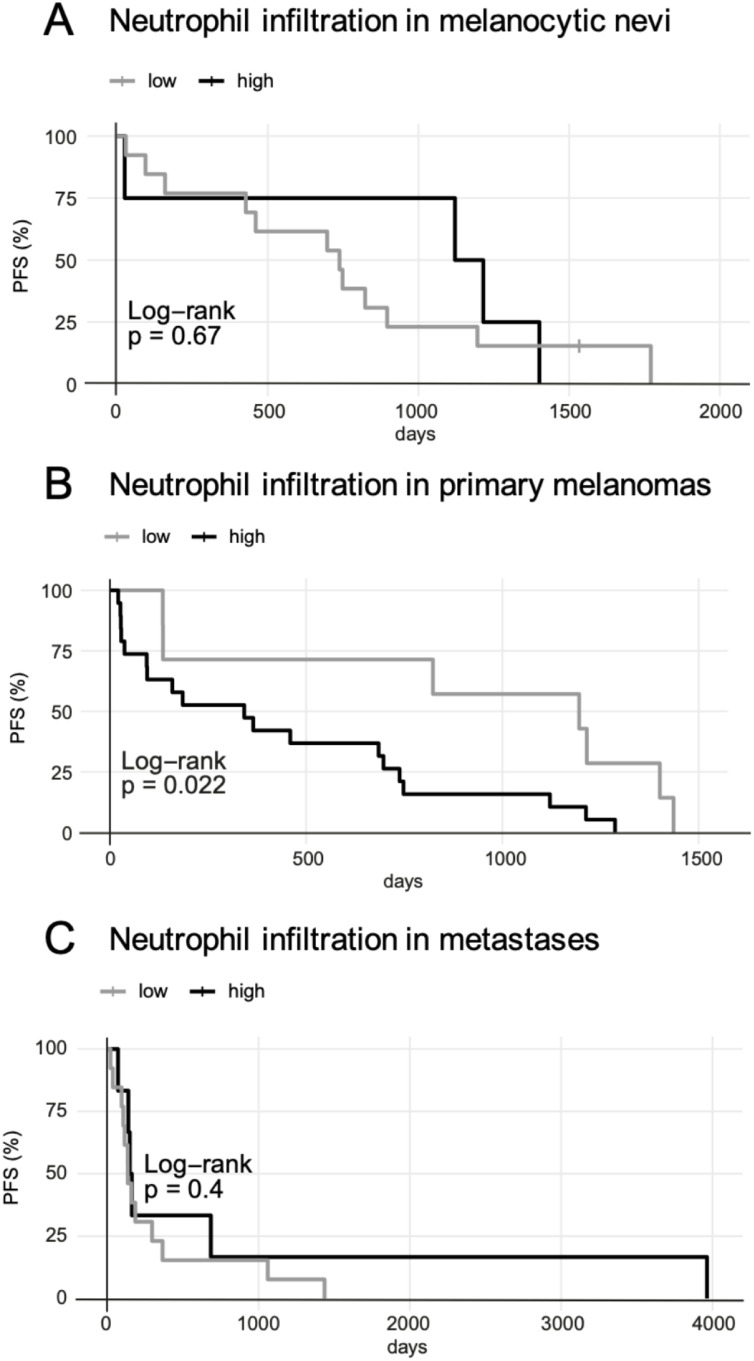
Univariate survival analyses: impact of neutrophil infiltration on progression-free survival (PFS) within melanocytic nevi, primary melanomas and metastases. Kaplan-Meier curves are shown for the melanoma patients in the tissue study. A) No significant difference in PFS was shown for high or low neutrophil infiltration in melanocytic nevi. Patients were stratified by high and low neutrophil infiltration in their melanocytic nevus. The "number at risk" at day 0 for CD15=low was 13 patients; for CD15=high, 4 patients. The cut-off value was 0/mm^2^. B) High neutrophil infiltration in primary melanomas could be associated with shortened PFS. Patients were stratified by high and low neutrophil infiltration in their primary melanoma. The "number at risk" at day 0 for CD15=low was 7 patients; for CD15=high, 19 patients. The cut-off value was 0.99/mm^2^. C) No significant difference in PFS was shown for high or low neutrophil infiltration in metastases. Patients were stratified by high and low neutrophil infiltration in their metastasis. The "number at risk" at day 0 for CD15=low was 13 patients; for CD15=high, 6 patients. The cut-off value was 6.27/mm^2^.

Hereafter, we seek to investigate the influence of neutrophils in the tumor microenvironment on tumor progression by means of survival analyses. In this study, OS was not suitable as endpoint of the Kaplan-Meier analyses, since hardly any deaths had occurred in the patient collective during the observation period. Therefore, only PFS was considered. An analysis within each tumor type was performed. The evaluation of the melanocytic nevi yielded no significant differences in PFS between patients with a pronounced neutrophil infiltrate in their melanocytic nevi and patients with melanocytic nevi showing lower neutrophil infiltration (see Fig. 3A). Furthermore, neutrophil accumulation in primary melanomas was correlated with PFS (see Fig. 3B). High neutrophil infiltration in primary melanomas could be associated with a shortened PFS (cut-off 0.99/mm^2^, *p* < 0.05). This means if numerous neutrophils were found in a patient's primary melanoma, tumor progression was likely to occur more rapidly compared to patients whose primary melanomas were infiltrated by fewer neutrophil granulocytes. Thirdly, the same evaluation was performed within the metastasis group. Here, we found no significant differences in terms of PFS between the group with high and the one with low neutrophil infiltration in metastatic lesions (see Fig. 3C).

Taken together, the prognostic significance of a high neutrophil infiltration in primary melanomas should be emphasized. It was associated with a shortened PFS in melanoma patients. This reflects tumor-promoting effects of neutrophils within the TME.

## Discussion

In this retrospective immuno-monitoring study, we sought to address the question if S100A8/A9 and neutrophils were reliable predictive biomarkers of clinical outcome in advanced melanoma patients. It has been demonstrated in previous research that ICIs like ipilimumab, nivolumab and pembrolizumab, and treatment with combinations thereof, can effectively prevent immune escape mechanisms, among which CD8^+^ T cell suppression is of paramount importance [34,42]. In this way, part of melanoma patients suffering from metastatic disease achieve prolonged survival [21,35]. However, another part of treated patients are never-responders, and a major percentage does only benefit from therapy during a limited time period [29]. We hypothesized that the secretion of immunosuppressive factors like S100A8/A9 by neutrophils could contribute to therapy failure via CD8 + T cell inhibition. This mechanism may play a role in the establishment of a “cold” TME.

Firstly, we analyzed S100A8/A9 as blood-based follow-up parameter for disease progression before and during immunotherapy because as such it seemed to be of major relevance for therapy decisions in clinical practice. We observed a significant negative effect of increased serum S100A8/A9 concentrations at baseline and during ICIs administration on PFS. This result is in line with previous research demonstrating that ipilimumab-non-responders showed increased S100A8/A9 serum levels after the first administration of the therapeutic drugs [15]. DAMPs such as S100A8/A9 were detected in the cytoplasm of activated neutrophils and are released during NETosis, i.e., a form of programmed cell death [10,38,45]. Especially Perego et al. confirmed that neutrophils release S100A8/A9 upon cellular stress and that higher concentrations of serum S100A8/A9 were associated with shorter time to recurrence in lung cancer [30]. In support of the concept that neutrophils are the main source of S100A8/A9, we performed immunofluorescence stainings of CD15 and S100A8/A9 in tissues of primary melanomas and healthy skin as control (see Supplements Fig. S1). We found a strong colocalization of neutrophils and S100A8/A9. Importantly, a strong increase of neutrophils expressing S100A8/A9 in primary melanomas was detected (see Supplements Fig. S1B and C) when compared to healthy skin as control (see Supplements Fig. S1A and C). N2-type TANs were shown to promote tumor progression by NET release [27,52]. In fact, S100A8/A9 represents a major component of NETs as previous studies have demonstrated [39,44]. In order to examine NETosis in the tumor microenvironment, we stained for citrullinated histone H3 (citH3). Fig. S2, which is depicted in the Supplements, shows that compared to control skin, neutrophils within primary tumor tissue released an increased number of NETs. In conclusion, our results provide additional evidence that neutrophil activation followed by the secretion of NETs in combination with the release of S100A8/A9 constitute potential biomarkers for prediction of the prognosis in melanoma patients. Due to the apparent link between S100A8/A9 and neutrophils, we dealt with the influence of systemic neutrophilia on tumor progression via Kaplan-Meier estimation. According to this analysis, PFS was shortened if neutrophil concentrations were high before starting the treatment regime and during the course of therapy. In this vein, previous research found a negative prognostic value of high circulating neutrophil concentrations before therapy initiation already [8,15]. By contrast, the analyses we prepared here revealed a favorable influence on OS for patients showing increased neutrophils at baseline and no significant impact at measurement point T3 (during ICIs treatment). These findings indicate a subordinate potential of systemic neutrophil concentrations as biomarker in our patient cohort.

It was shown *in vitro* that a NET-rich TME induced a functional and metabolic exhausted phenotype in tumor-infiltrating T cells via PD1/PDL1 engagement [23]. This finding could also explain why S100A8/A9 served as a negative predictive biomarker in the context of our study. T cell inhibition does seem to be the crucial connecting piece between increased neutrophil-lymphocyte ratio (NLR) and tumor progression. Yet, it remains to be explored if S100A8/A9 plays a direct functional role in T cell suppression or if S100A8/A9 is only indicative of NETosis-mediated T cell anergy. As our experiments so far included mainly circulating neutrophils in the peripheral blood, we were further interested in the role of TANs. We thus performed immunofluorescence stainings of neutrophils in melanocytic nevi, primary melanomas and metastases. In our analysis of 292 tumor tissue samples, we found significant differences between neutrophil accumulation in the three tumor types examined. The smallest number of neutrophils was detectable in melanocytic nevi and the most extensive tumor tissue neutrophilia was found in primary melanomas. It has been suggested in the literature that the number of tumor-infiltrating neutrophils increases during melanoma progression [37]. Intuitively, we would have expected to find more (rather than less) neutrophils in metastases compared to primary melanomas. However, it seems to be consistent with the research on NETosis that fewer intact neutrophils could be stained in metastatic lesions [10]. Indeed, this finding might be explained through an altered functional state of neutrophils in metastases and them bursting in NETosis upon their activation. In various publications, it was demonstrated that an increased number of neutrophil granulocytes in the tumor bed was associated with a significantly worse prognosis [22,31,41]. In order to add more data besides our expression analysis, we performed a univariate survival analysis regarding the effect of high counts of TANs on prognosis. The most prominent finding was that an extensive neutrophil infiltration in primary melanomas correlated with reduced PFS. Various studies have demonstrated that NETs contained in neutrophils are pivotal for their pro-tumorigenic effects [5,26,43]. Novel combination regimens could be developed in order to inhibit neutrophil NETosis and thereby S100A8/A9 release. Several approaches have been developed to target NETosis in preclinical cancer models such as removal of NETs using DNase I, inhibition of the enzyme peptidylarginase deiminase 4 (PAD4) through GSK484, or blockade of the CXCR1/2 axis with reparixin [3,7,16,33,50]. In order to explore these opportunities further, the role of tissue-resident S100A8/A9 in NET-rich tumor beds needs to be characterized more in detail. Moreover, it would be interesting to test the correlation between the amounts of neutrophils and CD8+ T cells infiltrating melanoma lesions. Since other players than S100A8/A9 may mediate the crosstalk between neutrophils and CD8+ T cells, the content of neutrophil granules should be further examined. Within the array of cytoplasmatic contents being released during NETosis, NADPH-oxidase, ROS, NO, NE and MPO may be essential for T cell immunoparalysis [6,9,51]. Apart from the effects of mediators extruded through NETosis, NET structures can physically shield tumor cells from CD8+ T cells and therefore protect them from cytotoxicity [40]. This discovery represents a glimpse into the complexity of NET biology and its relevance for antitumor immunity. It remains to be explored specifically in melanoma.

In summary, S100A8/A9 and neutrophils seem to serve as predictive markers in combination with other markers. Further studies in larger cohorts of ICIs-treated patients are needed in order to proof whether high serum S100A8/A9 and neutrophil counts correlate with neutrophil migration into the tumor tissue, their specific cellular roles in the tumor microenvironment, and their possible use as biomarkers for treatment monitoring.

## Conclusions

Our results highlight that an increased level of circulating S100A8/A9 during ICIs treatment impair clinical outcome of metastatic melanoma patients. Moreover, the accumulation of TANs in primary lesions correlates with impaired survival. These findings underline the relevance of neutrophils and S100A8/A9, which is secreted by stressed neutrophils upon NETosis, as drivers of melanoma progression. Specifically, neutrophils could affect response to ICIs by contributing to CD8+ T cell dysfunctionality.

In clinical practice, serum S100A8/A9 and neutrophil counts could serve as follow-up biomarkers being measured in sequential liquid biopsies during ICIs administration. In this way, they might provide additional clinical value in disease monitoring. Moreover, our study warrants for testing therapies targeting S100A8/A9 and other NET-associated factors linked to neutrophils’ immunosuppressive properties in metastatic melanoma.

## Institutional review board statement

The study was conducted in accordance with the Declaration of Helsinki, and approved by the Hamburg Ethics Committee (PV5392 on 6th December 2016) and by the Ethics Committee II of Heidelberg University (2010-318N-MA and 2014-835R-MA).

## Informed consent statement

Written informed consent was obtained from all subjects involved in the study.

## CRediT authorship contribution statement

**Yasmin F Melzer:** Writing – review & editing, Writing – original draft, Visualization, Validation, Software, Methodology, Investigation. **Nadine L Fergen:** Writing – review & editing, Investigation. **Christian Mess:** Writing – review & editing, Validation, Software, Methodology, Formal analysis. **Julia-Christina Stadler:** Writing – review & editing, Validation, Resources. **Glenn Geidel:** Writing – review & editing, Resources, Investigation. **Ysabel A Schwietzer:** Writing – review & editing, Methodology. **Julian Kött:** Writing – review & editing, Resources, Methodology. **Klaus Pantel:** Writing – review & editing, Supervision, Resources. **Stefan W Schneider:** Writing – review & editing, Supervision, Resources. **Jochen Utikal:** Writing – review & editing, Resources. **Ewa Wladykowski:** Investigation. **Sabine Vidal-y-Sy:** Investigation. **Alexander T Bauer:** Writing – review & editing, Writing – original draft, Supervision, Methodology, Investigation, Conceptualization. **Christoffer Gebhardt:** Writing – review & editing, Writing – original draft, Visualization, Validation, Supervision, Software, Resources, Project administration, Methodology, Investigation, Funding acquisition, Formal analysis, Data curation, Conceptualization.

## Declaration of competing interest

The authors declare the following financial interests/personal relationships which may be considered as potential competing interests: C.G. is on the advisory board or has received honoraria from Almirall, Amgen, Beiersdorf, BioNTech, Bristol-Myers Squibb, Delcath, Immunocore, Janssen, MSD Sharp & Dohme, Novartis, Pierre-Fabre Pharma, Roche, Sanofi Genzyme, SUN Pharma and Sysmex/Inostix, research funding from Novartis, Regeneron and Sanofi Genzyme, and travel support from Bristol-Myers Squibb, Pierre Fabre Pharma and SUN Pharma, outside the submitted work. C.G. is co-founder of Dermagnostix and Dermagnostix R&D. J.U. is on the advisory board or has received honoraria and travel support from Amgen, Bristol Myers Squibb, GSK, Immunocore, LeoPharma, Merck Sharp and Dohme, Novartis, Pierre Fabre, Roche, Sanofi outside the submitted work. All of the other authors declare no conflict of interest.

## Data Availability

For original data please contact ch.gebhardt@uke.de.

## Appendix Supplementary materials

Image, application 1
