# Supplementary material for: Evaluation of S100A8/A9 and neutrophils as prognostic markers in metastatic melanoma patients under immune-checkpoint inhibition
Source: Transl Oncol. 2024 Dec 18;52:102224. doi: 10.1016/j.tranon.2024.102224 (PMC11718343; doi:10.1016/j.tranon.2024.102224)
Supplement: Supplementary file 1 [file mmc1.docx]

*Supplements*

Evaluation of S100A8/A9 and Neutrophils as Prognostic Markers in Metastatic Melanoma Patients under Immune-Checkpoint Inhibition

*Yasmin F Melzer, Nadine L Fergen, Christian Mess, Julia-Christina Stadler, Glenn Geidel, Ysabel A Schwietzer, Julian Kött, Klaus Pantel, Stefan W Schneider, Jochen Utikal, Ewa Wladykowski, Sabine Vidal-y-Sy,* *Alexander T Bauer, Christoffer Gebhardt*

**Table S1.** Blood-based analysis: baseline and T3 serum levels (S100A8/A9, neutrophils)

| **Parameter** | **CR**  **(n=11)** | **PR**  **(n=16)** | **SD**  **(n=5)** | **PD**  **(n=11)** | **Total**  **(n=43)** |
| --- | --- | --- | --- | --- | --- |
| **S100A8/A9 (ng/ml)** |  |  |  |  |  |
| Baseline: mean (sd) | 2606.9 (1376.7) | 5350.3 (3566.6) | 3009.7 (1187.9) | 4030.0 (1546.4) | 4038.6 (2648.9) |
| T3: mean (sd) | 2573.9 (1009.3) | 4052.3 (2230.2) | 2239.0 (925.1) | 3824.7 (1545.6) | 3405.0 (1793.8) |
| **Neutrophils (/µl)** |  |  |  |  |  |
| Baseline: mean (sd) | (n=11)  4.4 (0.7) | (n=13)  5.0 (1.2) | (n=4)  4.9 (0.4) | (n=9)  5.3 (1.8) | (n=37)  4.9 (1.2) |
| T3: mean (sd) | (n=8)  4.7 (3.3) | (n=10)  5.5 (2.3) | (n=5)  4.5 (1.2) | (n=8)  5.9 (3.1) | (n=31)  5.2 (2.6) |

Abbreviations: sd: standard deviation. n: number.

**Table S2.** Tissue-based analysis: Descriptive parameters

| **Parameter** | **Melanocytic nevi**  **(n=44)** | **Primary melanomas**  **(n=86)** | **Metastases**  **(n=162)** | **Total**  **(n=292)** |
| --- | --- | --- | --- | --- |
| **CD15** |  |  |  |  |
| total (signal counts) | 78 | 3795 | 2448 | 6321 |
| **Punch area** |  |  |  |  |
| total (mm^2^) | 8,394,524.56 | 49,064,194.7 | 833,983,305.0 | 891,442,025.0 |
| **CD15 (normed per area)** |  |  |  |  |
| mean (sd) | 7.0 (26.0) | 81.2 (249.7) | 28.0 (129.7) | 40.4 (168.3) |

Abbreviations: sd: standard deviation. CD: cluster of differentiation. n: number


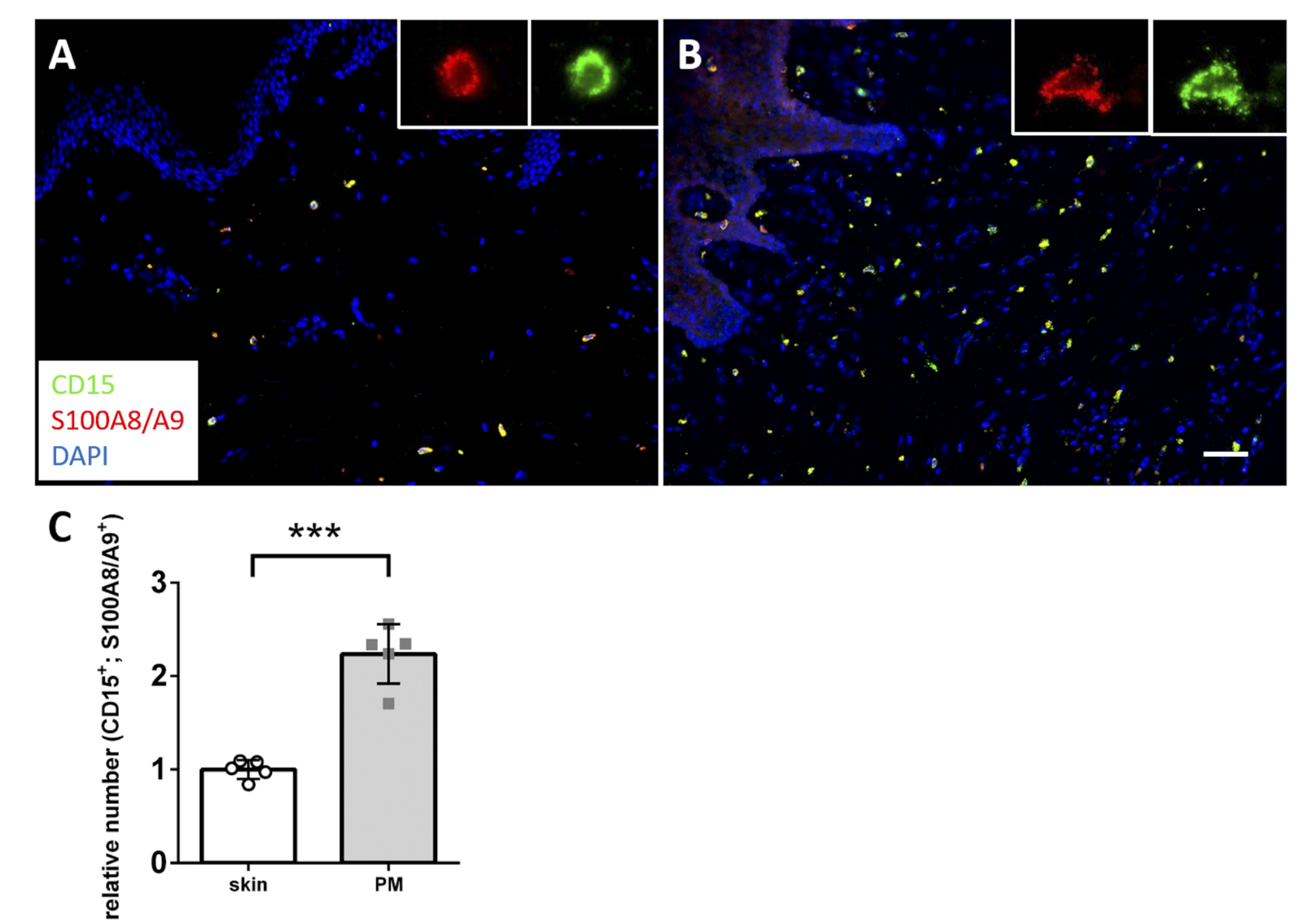


**Figure S1.** Immunofluorescence stainings and analyses of neutrophils and S100A8/9 in healthy skin (**A**) and in primary melanomas (**B**). Statistical analysis of all cell counts obtained (**C**). Shown are representative images of the tissue. The tissue samples were stained with an anti-CD15 antibody in conjunction with the secondary antibody Alexa 594 to visualize neutrophil granulocytes in green color and an anti-S100A8/9A antibody in conjunction with the secondary antibody Alexa 555 in red color. A single neutrophil from each punch is shown magnified. Blue nuclear counterstaining was performed with DAPI. Graphically, the mean ± standard deviation of the total 5 healthy skin controls and 5 primary tumors examined are listed in C. Significance levels: ***: p < 0.001. Abbreviations: PM: primary melanoma. Scale bar: 50 µm.


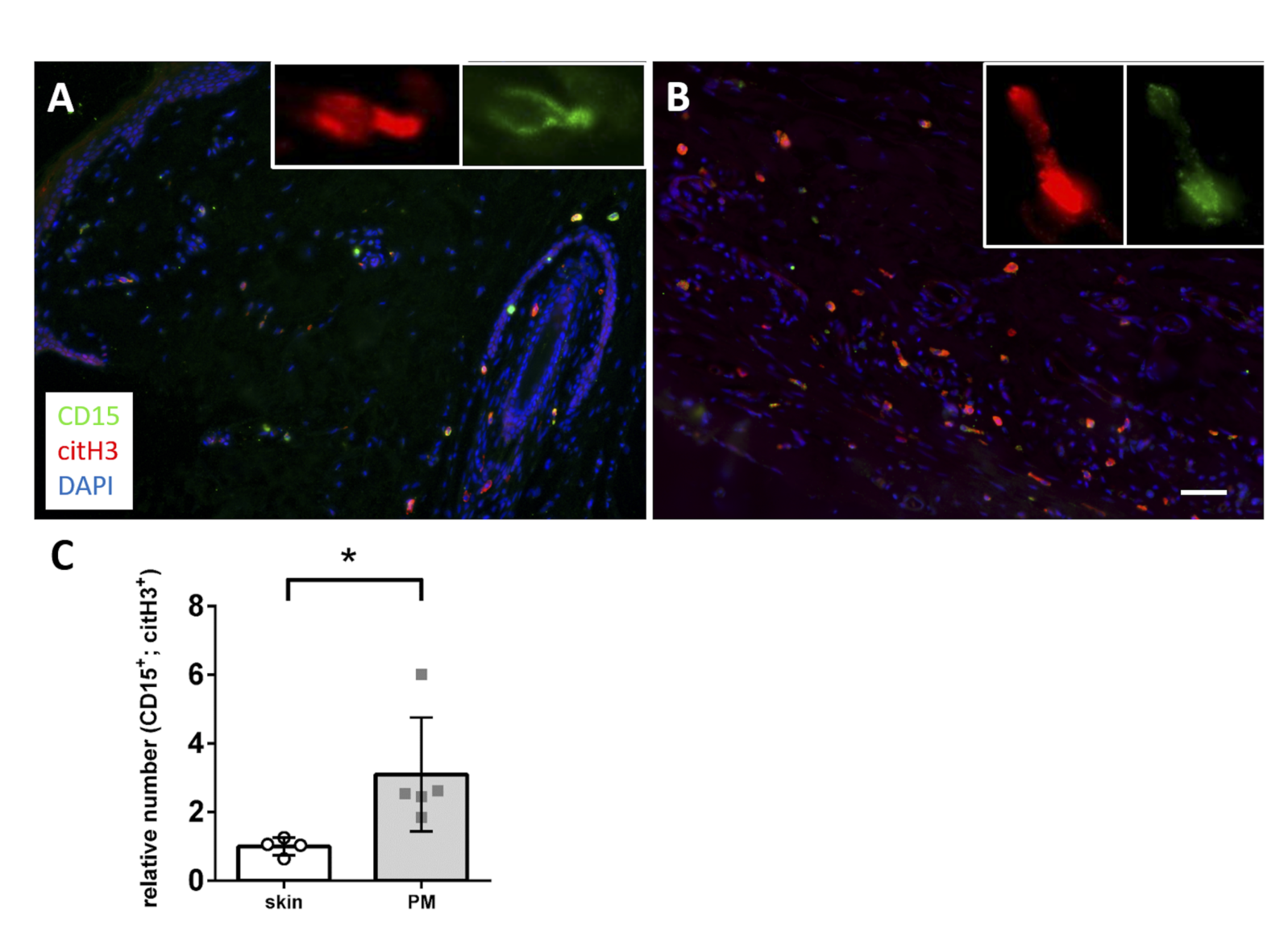


**Figure S2**. Immunofluorescence stainings and analyses of neutrophils and citrullinated histone H3 (citH3) in healthy skin (A) and in primary melanomas (B). Statistical analysis of all cell counts obtained (C). Shown are represantative images of the tissue. The tissue samples were stained with an anti-CD15 anti-body in conjunction with the secondary antibody Alexa 594 to visualize neutrophil granulocytes in green color and an anti- citH3 antibody in conjunction with the secondary antibody Alexa 555 in red color. A single neutrophil from each punch is shown magnified. Blue nuclear counterstaining was per-formed with DAPI. Graphically, the mean ± standard deviation of the total 4 healthy skin controls and 5 primary tumors examined are listed (C). Significance levels: *: p < 0.05. Abbreviations: PM: primary mel-anoma. Scale bar: 50 µm.
